# Exploring the binding sites and binding mechanism for hydrotrope encapsulated griseofulvin drug on γ-tubulin protein

**DOI:** 10.1371/journal.pone.0190209

**Published:** 2018-01-11

**Authors:** Shubhadip Das, Sandip Paul

**Affiliations:** Department of Chemistry, Indian Institute of Technology Guwahati, Guwahati, Assam, India; UMR-S1134, INSERM, Université Paris Diderot, INTS, FRANCE

## Abstract

The protein *γ*-tubulin plays an important role in centrosomal clustering and this makes it an attractive therapeutic target for treating cancers. Griseofulvin, an antifungal drug, has recently been used to inhibit proliferation of various types of cancer cells. It can also affect the microtubule dynamics by targeting the *γ*-tubulin protein. So far, the binding pockets of *γ*-tubulin protein are not properly identified and the exact mechanism by which the drug binds to it is an area of intense speculation and research. The aim of the present study is to investigate the binding mechanism and binding affinity of griseofulvin on *γ*-tubulin protein using classical molecular dynamics simulations. Since the drug griseofulvin is sparingly soluble in water, here we also present a promising approach for formulating and achieving delivery of hydrophobic griseofulvin drug via hydrotrope sodium cumene sulfonate (SCS) cluster. We observe that the binding pockets of *γ*-tubulin protein are mainly formed by the H8, H9 helices and S7, S8, S14 strands and the hydrophobic interactions between the drug and *γ*-tubulin protein drive the binding process. The release of the drug griseofulvin from the SCS cluster is confirmed by the coordination number analysis. We also find hydrotrope-induced alteration of the binding sites of *γ*-tubulin protein and the weakening of the drug-protein interactions.

## Introduction

Cancer is defined as a group of diseases involving uncontrolled cell growth and the spread of cells that can affect to any other part of the body [1]. In recent times this disease spreads universally and it has appeared as one of the most dreaded miseries. Cancer causes about millions of death in every years. 8 million lives had been devoured by this disease in 2010. The occurrence of cancer increases by 38% since the last 20 years [2]. According to the world cancer report, around 14.1 million new cases of cancer arise worldwide [3]. As per the report of GLO-BOCAN, by 2030, the global burden is expected to grow to 21 million new cancer cases [4].

**Fig 1:**
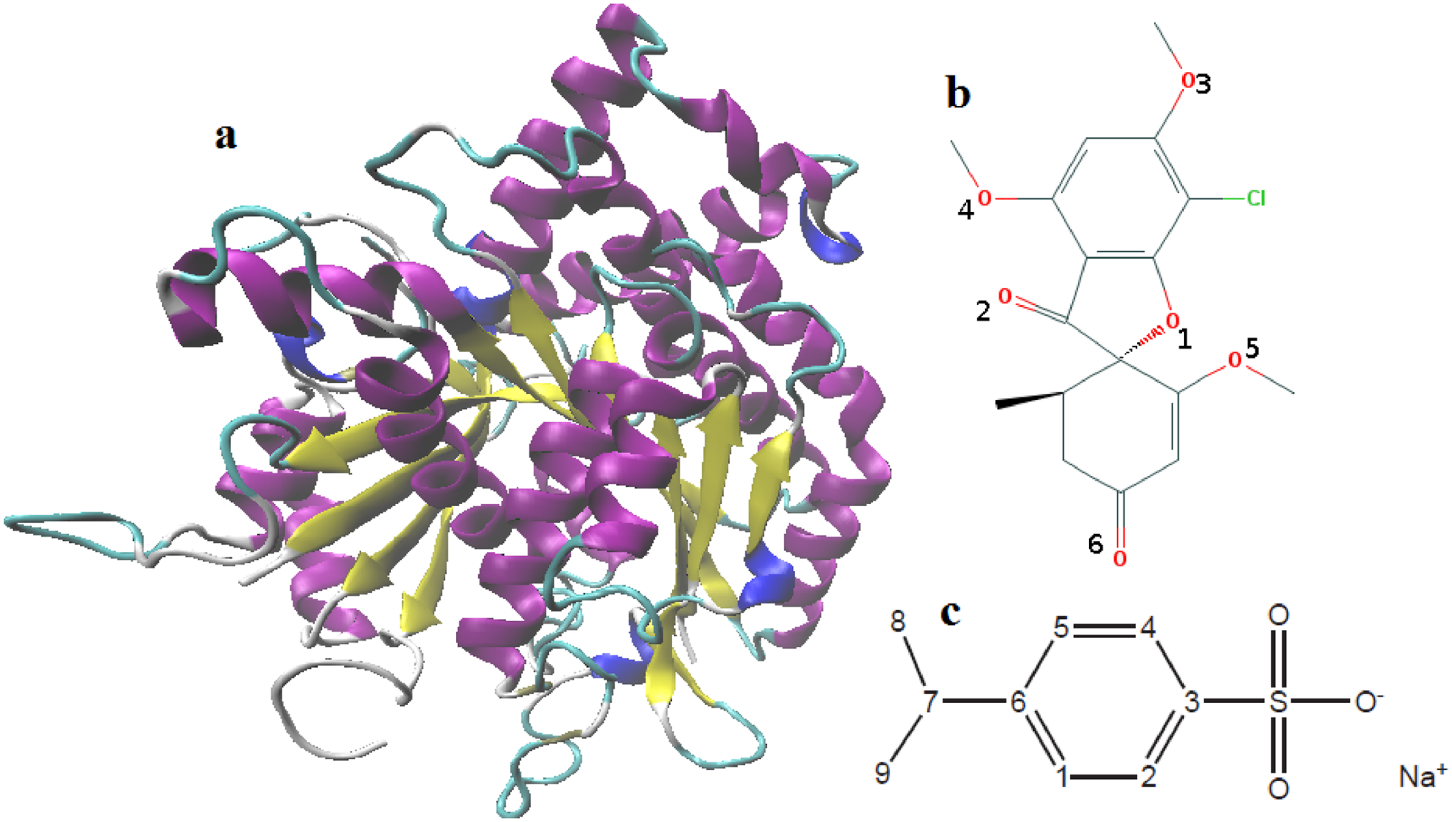
(a) Secondary structure of *γ*-tubulin protein, (b) structure of griseofulvin molecule and atomic number of oxygen atoms and (c) structure and atomic numbers of sodium cumene sulfonate. Hydrogen atoms are left off for better clarity in structures (b) and (c).

Centrosome commonly alludes to as polar corpuscle. In animal cell it functions as the microtubule organizing center. During kinesis it can administer spindle formation that assured a uniform distribution of genome [5]. Normal functioning of centrosome and successful spindle formation of non-transformed cells is controlled by the gamma tubulin (*γ*-tubulin) protein (See Fig 1 (a)), centrin etc [6]. The presence of multiple centrosome causes the multipolar spindle formation that direct the cell toward apoptosis. Presence of multiple centrosome within the same cell create a condition that is often referred to as supernumerary centrosomes. It is a crucial indication of most form of cancers [7]. The general scheme of cells with supernumerary centrosomes to persevere and cause destruction by its demonstration as cancer is its capability to experience a phenomenon called as centrosomal clustering or centrosome clustering [8]. Thus, it becomes very important to recognize the centrosomal clustering process to prepare the strategies for its destruction. The long search for inhibitors that can inhibit the centrosomal clustering is still in its early stage of development. There are numbers of natural origin compounds reported previously that can inhibit centrosomal clustering in tumor cell both in vitro as well as in vivo [7]. In an attempt to inhibit the centrosomal clustering, the inhibitors attack the main molecular targets, which are: (1) tubulin polymerization, (2) cell cycle regulation, (3) spindle tension, and (4) ability to affect mitotic spindle. In order to get a polarized, radial array of microtubules, localization of *γ*-tubulin to the spindle is necessary that helps to reorganize minus end of microtubule [9]. This process confirms the natural cell divisions [10]. Hence, an effective way to inhibit the centrosomal clustering is to target the *γ*-tubulin protein.

Griseofulvin is a lipophilic orally active fungi-static antibiotic drug (See Fig 1 (b)) [11]. Recently, griseofulvin has been attracted a significant interest as a potential anticancer drug because of its low toxicity and has an efficiency to inhibit the proliferation of various types of cancer cells [12–15]. It can also inhibit the growth of tumor in athymic mice [12]. It has ability to induce apoptosis in many cancer cell lines [16]. It has been observed that the griseofulvin can selectively kill the cancer cells without affecting any normal healthy cells [14]. It has also been reported that griseofulvin can inhibit the growth of fungal, plant and mammalian cells mostly by inducing unnatural mitosis and also by blocking the cells at G2/M phase of cell cycle [12–14, 17–19]. As griseofulvin shows different affinity to dissimilar tubulin, therefore, various organisms display non-identical degrees of sensitivity to griseofulvin [17, 20]. Since griseofulvin possesses much higher affinity towards fungal tubulin than the mammalian tubulin, so the concentration needed to inhibit the growth of fungal cells is much lower than the mammalian cells [17, 21–23]. Previously, it has been reported that griseofulvin can interact with tubulin [13, 23–27] and also with microtubule associated protein [24, 28]. Although, there are several studies on the *αβ*-tubulin [29, 30], suggested that tubulin is the primary target of griseofulvin but the binding mechanism, binding activity or the binding sites of novel anticancer drugs towards *γ*-tubulin is mostly unexplored.

According to the Biopharmaceutics Classification System griseofulvin possesses low solubility and high permeability character (ClassII drugs) [31]. ClassII drugs possess poor gastrointestinal tract solubility which basically limits their uses [32]. The problem of aqueous solubility can be solved by formulating the drug with nontoxic, water soluble molecules known as hydrotropes [33–36]. Hydrotropes are small organic amphiphilic molecules and they have resemblance in structural features with classical surfactant. They can self-aggregate in aqueous solution above minimum hydrotrope concentration (MHC) [37]. Most interestingly hydrotropes have an ability to solubilize the insoluble or sparingly soluble molecules in water [38, 39]. In our previous study we have shown the mechanism of hydrotropic action by formulating griseofulvin with hydrotrope sodium cumene sulfonate (SCS) molecules (See Fig 1 (c)) [39]. We found that SCS increased the solubility of griseofulvin by incorporating it within SCS clusters.

Here, in this study our aim is to understand the potential binding mechanism and binding sites for griseofulvin on *γ*-tubulin protein. Our particular interest in this study is that whether griseofulvin can bind with *γ*-tubulin protein if initially griseofulvin is in the cage of SCS clusters. We focus on this particular aspect because of the fact that griseofulvin alone (without any hydrotrope) is poorly soluble in water. Since, the presence of hydrotrope SCS molecules enhances its aqueous solubility [39] it would be interesting to look at: (i) in solution whether the drug griseofulvin is released from SCS cluster and (ii) how does the presence of SCS cluster affect the drug-protein interaction provided the condition (i) is fulfilled. To understand these we perform both all atom classical molecular dynamics simulation as well as drug-protein binding free energy calculations using MM-GBSA methods. We expect that the detailed understanding of the *γ*-tubulin and griseofulvin interactions provide some useful insights for designing better analogues in future.

The remaining of this paper is divided into four sections. Simulation and free energy calculation details are described in simulation and methods and the methodology of binding free energy calculations sections respectively. Then results are presented and discussed and in the last section our conclusions are outlined concisely.

## Models and simulation method

In this study classical molecular dynamics (MD) simulation was performed to investigate the binding ability of griseofulvin drug molecules to *γ*-tubulin protein in presence and in absence of hydrotrope SCS. The different systems considered in this study are tabulated in Table 1. The initial crystal structure of *γ*-tubulin was downloaded from the protein data bank (PDB ID 3CB2 [40, 41]). The Insight II graphics package [42] was used to add the coordinates of the missing residues of *γ*-tubulin. The hydrogens attached to heavy atoms were added by using the leap program of AMBER12 package [43]. To neutralize the negatively charged *γ*-tubulin, 15 Na^+^ counter ions were added using XLEAP of AMBER12 program. The model single chain of modified *γ*-tubulin consists of 474 amino acid residues. The AMBERff99 force field [44] parameters were adopted for the different atomic sites of the protein molecule. For the different atomic sites of the drug griseofulvin, we used the force field parameters and the partial charges that were developed in our earlier work [39]. CHARMM General Force Field (CGenFF) was adopted for SCS molecules [45, 46]. To counterbalance the single negative charge carried by the sulfonate group of SCS molecule, one Na^+^ counter ion was added using the XLEAP of AMBER12 package. The SPC/E model was chosen for the water molecules [47]. The stability of the protein in pure water was investigated by immersing the protein in water in absence of any cosolute molecules (system P0). Then three different systems were prepared that contain griseofulvin drug molecule in the cage of SCS clusters (systems P4-P6) and three without SCS aggregates (systems P1-P3). In order to prepare the griseofulvin encapsulated SCS clusters, following our recent works [39], initially a system was constructed that consisted of 24 hydrotrope SCS molecules and one griseofulvin molecule (in 24:1 ratio). At first all the SCS and griseofulvin molecules were placed randomly. The initial configuration of the hydrotrope-drug mixture was equilibrated in the vacuum. The vacuum simulation run was carried out in AMBER12 for 12 ns at 298 K with a time step of 2 fs. In vaccum the hydrotrope molecules formed reverse micelle kind of structure and griseofulvin molecule was found at the surface of reverse micelle structure (S1a and S1b Fig). Next, a cubic box around the compacted complex was generated with the help of leap module of AMBER12 using 0 Å buffer constant in all the three directions. The voids mainly created at the corners of the cubic box, were filled up by 405 SPC/E water molecules. Then, energy of the system was minimized for 10000 steps, where first 4000 steps were performed in steepest descent minimization method and this was followed by another 6000 steps in conjugate gradient method. Thereafter, the system was heated slowly from 0 K to 298 K in a canonical ensemble (NVT). Finally, the system was subjected to 24 ns equilibration in isothermal isobaric (NPT) ensemble at 298 K and 1 atm pressure. At the end of the simulation the hydrotrope molecules adopt a micelle like structure where small hydrophobic part of SCS aggregates around the griseofulvin molecule, whereas the hydrophilic part of SCS molecule is directing towards water molecules [39] (S1c and S1d Fig). From the last step of the simulated trajectory, the coordinates for SCS and griseofulvin molecules were extracted. Finally, after checking the insulation of the drug in the cage of hydrotrope cluster, these coordinates were used to prepare the systems P4-P6 for this study. In the initial starting configurations, the *γ*-tubulin and griseofulvin molecules were placed randomly in close contact for the systems P1-P3. In the same way, the protein and griseofulvin containing SCS cluster were placed randomly closed to each other in systems P4-P6. In each and every simulations, protein was fixed at the center of the simulation box. Therefore, a total of 7 MD simulation runs were performed with the AMBER12 package at 298 K temperature and 1 atm pressure. Using PACKMOL software [48], the initial configurations of each of these systems was constructed and the molecules were placed in a cubic box. For all the systems periodic boundary conditions were applied in all the three directions. For all the systems, to obtain proper initial structures following the methods stated above, 10000 steps of energy minimization were performed. Note that during the energy minimization, two-step minimizations were performed to relieve bad van der Waals contacts. At first the protein was held fixed by using harmonic restraints (force constant = 6000.0 kcal mol^−1^ Å^−2^) and then withdrawing the restraints on the protein. Subsequently, each of the energy minimized systems was then gradually heated from 0 K to 298 K in NVT ensemble for 320 ps using a 2 fs time step. The systems were then equilibrated for 25 ns and then the equilibrated structures were further simulated for 140 ns to generate production phase trajectories in NPT ensemble, at 298 K and 1 atm pressure. The SHAKE algorithm was used to constrain bonds involving hydrogen and heavy atoms [49]. During the simulation, the temperature of the simulation box was controlled by the Langevin dynamics method with a collision frequency of 1 ps^−1^. In addition, the pressure was controlled by Berendsen barostat [50] using a pressure relaxation time of 2 ps. The particle mesh Ewald method was used to treat the non-bonded long ranged electrostatic interactions and for all short-ranged nonbonded interactions a cut-off distance of 12.0 Å was used.

**Table 1:** Overview of systems.

System	*N*_*Protein*_	*N*_*GSV*_	*N*_*SCS*_	*N*_*Water*_	Box Volume(*nm*^3^)
P0	1			25000	812.95
P1	1	1		25000	814.78
P2	1	1		25000	813.89
P3	1	1		25000	815.22
P4	1	1	24	25000	821.68
P5	1	1	24	25000	821.44
P6	1	1	24	25000	822.08

*N*_Protein_, *N*_GSV_, *N*_*SCS*_ and *N*_*Water*_ are the number of *γ*-tubulin protein, griseofulvin drug, hydrotrope sodium cumene sulfonate, and water molecules respectively.

## Binding free energy calculations

In order to explore the differential binding affinity of *γ*-tubulin protein with griseofulvin drug molecule, for different systems binding free energies were calculated using MM-GBSA method [51]. Each of the MM-GBSA calculations was carried out with the help of Python script MMPBSA.py of AMBER12 package. The binding free energies for different systems were calculated using last 4 ns of the corresponding production phase trajectories. The free energy of complex, receptor and ligand can be estimated using the series of following equations [30]:

$$\begin{array}{c} \Delta G_{bind}=G_{complex}-G_{receptor}-G_{ligand} \end{array}$$ (1)

The Δ*G*_*bind*_ (without entropic contribution) can be calculated from the changes in molecular mechanical energies (Δ*E*_*MM*_) and solvation free energy Δ*G*_*solv*_ as follows: [29]

$$\begin{array}{c} \Delta G_{bind}=\Delta E_{MM}+\Delta G_{solv} \end{array}$$ (2)

Δ*E*_*MM*_ is the sum of van der Waals (Δ*E*_*vdw*_) and electrostatic (Δ*E*_*ele*_) energies.

$$\begin{array}{c} \Delta E_{MM}=\Delta E_{vdw}+\Delta E_{ele} \end{array}$$ (3)

Δ*G*_*solv*_ can be calculated as [39]

$$\begin{array}{c} \Delta G_{solv}=\Delta G_{GB}+\Delta G_{NP} \end{array}$$ (4)

The polar component of free energy change (Δ*G*_*GB*_) was calculated by the use of generalized-Born (GB) approach [52]. On the contrary non polar part (Δ*G*_*NP*_) of the solvation free energy was determined by the use of following equation [52, 53]:

$$\begin{array}{c} \Delta G_{NP}=\gamma \left(SASA\right)+\beta \end{array}$$ (5)

where *γ* = 0.0072 kcal/Å^2^ and *β* = 0.0, and SASA is the solvent accessible surface area.

## Results and discussion

### Root mean square deviation (RMSD)

**Fig 2:**
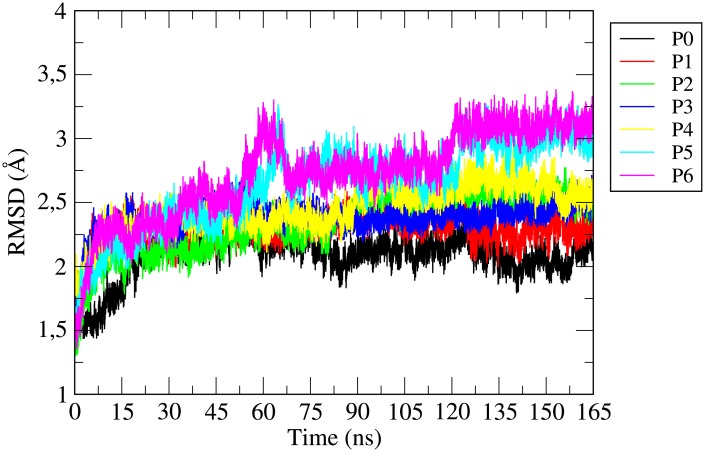
Variations of root mean square deviations (RMSDs) of the backbone *C*_*α*_-atoms of different residues of *γ*-tubulin protein as simulation progresses.

In order to examine the conformational stability of the protein during the MD trajectories *165ns*, the atom positional root mean square deviations (RMSDs) of the C_*α*_ backbone atoms of the residues of *γ*-tubulin protein are calculated for all the systems considered here and the same are shown in Fig 2. The downloaded original crystal structure of *γ*-tubulin 3CB2 does not contain C-terminal region in its template structure. Thus, the RMSD of *γ*-tubulin protein of the last 28 C-terminal amino acid region of *γ*-tubulin displays a large increased and more fluctuated RMSD. Therefore, the RMSDs (eliminating the C-terminal region) of C_*α*_ backbone atoms starting from amino acid GLY-446 are calculated. A comparison of the RMSD values of the different systems implies that the *γ*-tubulin protein undergoes structural changes and its conformation deviates significantly from the initial starting conformation. The relative fluctuation in the RMSD values of C_*α*_ atom of the protein is very small for all the systems once the equilibration is attained, indicating the convergence of simulations. Further, it can also be seen that the RMSD of *γ*-tubulin in pure aqueous system (P0) is almost similar with that of drug containing systems without SCS molecules (i.e., systems P1-P3), which indicates that the conformations of *γ*-tubulin in systems P1-P3 are almost similar to that for system P0. Concentrating on the systems with SCS-cluster encapsulated drug molecule (systems P4-P6) we observe a moderate deviation of the protein conformation from its initial conformation. Therefore, *γ*-tubulin conformation has achieved more stability in the drug bound states in absence of SCS molecules than the drug bound protein in presence of SCS molecules. Since, in order to check the reliability of our results, we have simulated the same system composition three times with different initial configurations and in all the cases excellent agreements have observed indicating the results presented here are trustworthy.

### Radius of gyration

**Fig 3:**
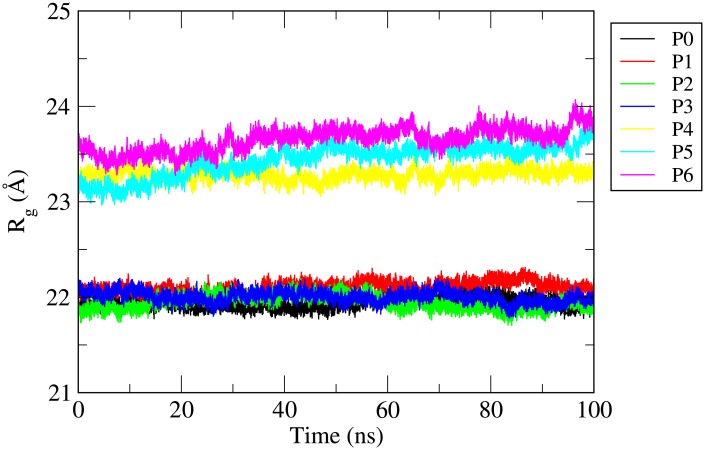
Radius of gyration (*R*_*g*_) of *γ*-tubulin in different systems versus simulation time.

To get an idea about the compactness of *γ*-tubulin structure, we have further determined the radius of gyration (R_*g*_) of *γ*-tubulin during the production phase MD trajectories for all the systems. R_*g*_ values actually give information about the size of the protein and that can be employed to achieve an insight to the stability of a protein throughout MD simulation trajectory. Fig 3 displays the R_*g*_ values versus simulation time of *γ*-tubulin both in pure water (system P0) and in drug bound state (systems P1 to P6). It is observed that R_*g*_ values are stable during the simulation for all the systems. So the results basically confirm that the overall compactness and stability of *γ*-tubulin protein are maintained during the simulation. A comparison of all seven R_*g*_ values show that the most packed *γ*-tubulin structures are acquired when protein is bound with griseofulvin in absence of hydrotrope SCS molecules (systems P1-P3) and in pure aqueous system (system P0). The moderately higher R_*g*_ values of the protein for the systems P4-P6 suggest the expansion of protein conformation in the presence of hydrotrope molecules. It may be because of the fact that some of the SCS molecules bind with the protein leading to a change in the protein conformation in systems P4-P6.

### Solvent accessible surface area (SASA)

The solvent accessible surface area (SASA) provides quantitative estimation of the surface area of the solute protein that is available to the solvent molecules. Using the Visible Molecular Dynamics (VMD) software [54], the SASA values of *γ*-tubulin protein for all the systems are calculated by considering a spherical probe radius of 1.4 Å throughout the protein surface. In Table 2 we have shown the average SASA values for all the systems. In the parentheses of the same table we have also included the standard errors of different SASA values. These standard errors are calculated by dividing the total simulation runs into five equal blocks. Then by considering independent SASA value of each block the standard errors are estimated. The first block values of SASA for systems P0-P6 are 21080.60, 21444.48, 21148.88, 21726.50, 26557.54, 26666.93 and 26895.00 Å ^2^ respectively. It is apparent that the maximum SASA value is achieved by the protein *γ*-tubulin for the system P6 and its smallest value is acquired for the system P2. The smaller SASA values for the systems P0-P3 than the systems P4-P6 indicate that the protein experiences more solvent exposure in presence of hydrotrope molecules. These findings imply that SCS molecules deliver some flexibility to protein conformation that ultimately leads to a possible expansion in *γ*-tubulin conformation. These observations are in accordance with the estimated RMSD and R_*g*_ values of different systems discussed above.

**Table 2:** Solvent accessible surface area (SASA) for different systems.

System	*SASA* (Å ^2^)
P0	21713.26 (±59.01)
P1	21717.26 (±59.00)
P2	21389.54 (±72.49)
P3	21569.55 (±59.44)
P4	26445.36 (±59.87)
P5	26702.29 (±41.59)
P6	26984.05 (±70.94)

The numbers inside the parentheses represent standard errors that are estimated by dividing the total simulation runs into five independent blocks (see text for details).

### Root mean square fluctuation (RMSF)

**Fig 4:**
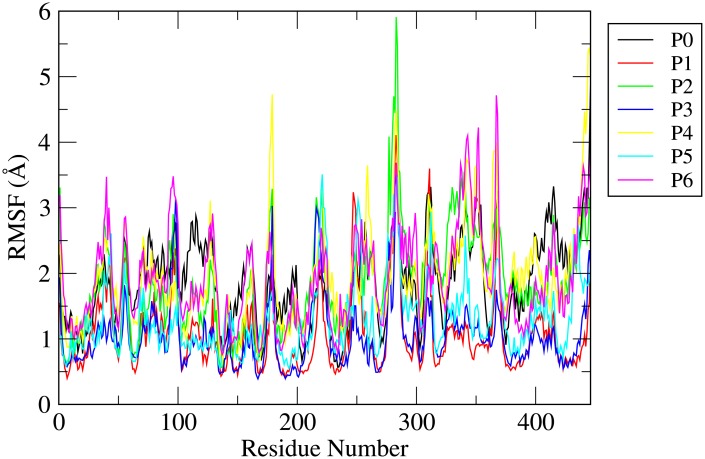
Root mean square fluctuations (RMSFs) of *C*_*α*_-atoms of all residues of *γ*-tubulin for different systems.

Root mean square fluctuation (RMSF) gives important information about the local structure transformation of each residue of *γ*-tubulin protein. RMSF actually determines the amount of deviation of C_*α*_ atoms of each residue from its average position. It is expected that the regions of the protein that are highly flexible will display high RMSF values whereas the constrained regions will exhibit low RMSF values. For different systems, RMSFs of all C_*α*_ atoms of *γ*-tubulin are calculated (see Fig 4). For all the systems the RMSFs of C_*α*_ atoms of all the residues of *γ*-tubulin fall within the range of 6.0 Å. Furthermore, it is clear that *γ*-tubulin protein in the drug bound state either in presence of SCS molecules or in absence of SCS molecules adopts very different dynamic behavior compared to that in pure aqueous solution. The fluctuations of RMSFs of C_*α*_ atoms of most of the residues of *γ*-tubulin for systems P1-P6 are higher than that of system P0. A comparison of different plots of RMSFs for different systems also suggests that the *γ*-tubulin displays higher fluctuations in presence of SCS molecules (systems P4-P6) than that for the systems devoid of SCS. The loop joining the S9 strand and H10 helix is very flexible. As our initial *γ*-tubulin protein PDB structure (3CB2) does not contain 278-283 residues, therefore this loop (residue 274-289) exhibits more flexibility. Again, the opening of this loop also helps the griseofulvin drug molecule to penetrate into the binding sites that also makes it more flexible. From Fig 4, it is also apparent that loops joining the S7 strand and H6 helix, S13 and S14 strands, and H11 helix and S13 strand also experience increased flexibility. It is also observed that the loops joining the H7 and H8 helices, H8 and H9 helices, and S10 and S11 strands display moderate flexibility. The adjacent regions i.e., H6, H7, H8, H9, H11 helices and S7, S11, S13, S14 strands also show significant flexibility. Therefore, it can be proposed that the functionally relevant loops and adjacent helices and strands can act as drug binding sites and exhibit large RMSF values.

**Fig 5:**
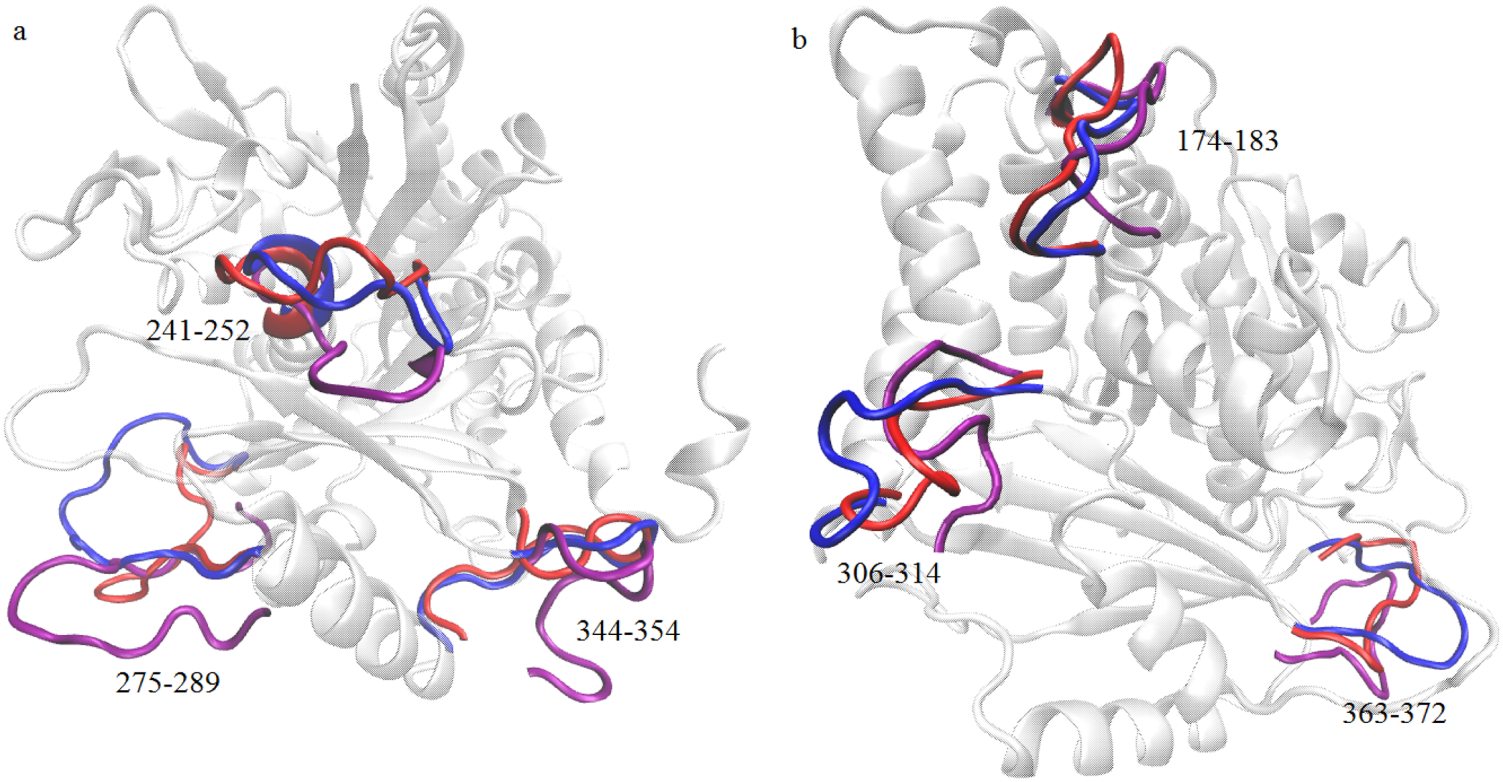
Structural changes of *γ*-tubulin with respect to system P0. Time averaged structures of *γ*-tubulin of systems P3 and P6 are superposed on the time averaged structure of *γ*-tubulin of system P0. To maintain the clarity of the picture, the most significant confomational changes of *γ*-tubulin protein are highlighted in both the structure (a) and (b). Blue, red and purple colors refer to the different sites of the protein *γ*-tubulin for systems P0, P3 and P6 respectively.

In Fig 5, we have presented a detailed structural comparison of *γ*-tubulin for the systems P0, P3 and P6. We created this figure by stereo superposition of the averaged structure of the protein for the systems P0, P3 and P6. We have considered these three systems because of the fact that one system is pure aqueous protein solution (system P0), one system contains only drug molecules (system P3) and the third one consists of encapsulated drug molecule by hydrotrope SCS. By considering the C_*α*_ atoms of *γ*-tubulin protein the average structure is calculated from the last 12 ns of simulation trajectories. In this figure, we highlight only those *γ*-tubulin residues for which the most significant structural changes occur. Larger fluctuations are perceived in the functionally relevant residues of *γ*-tubulin for the systems P3 and P6 than that for the pure aqueous system i.e. system P0. The loops joining the S7 strand and H6 helix (residues 174-183), H8 and H9 helices (residues 241-252), S9 strand and H10 helix (residues 275-289), S10 and S11 strands (residues 306-314), H11 helix and S13 strand (residues 344-354), and S13 and S14 strands (residues 363-372), that are significant sites for drug binding (discussed later), exhibit maximum deviations for the systems P3 and P6. In pure aqueous system, the loops of *γ*-tubulin protein stay in a stable inward conformation while in other drug containing systems (systems P3 and P6) the loops extend to the outward conformation. Some other helices and strands also display small variations, which are not shown to maintain the visual clarity of the figure. We note that findings are in accordance with RMSF analyses discussed above.

### Coordination number

In order to facilitate the interaction between drug griseofulvin and different residues of the protein *γ*-tubulin, the former has to be released from inside of the SCS core. This can be captured qualitatively (and indirectly) by calculating the average number of first shell SCS molecules (first shell coordination number, *CN*) around the drug griseofulvin for different systems. The *CN*s can be estimated from the site-site pair-correlation function involving center of mass (*COM*) of griseofulvin and *C*_7_ atomic site of SCS molecules as:

$$\begin{array}{c} CN=4\pi \rho_{\beta }\int_{r_{1}}^{r_{2}}r^{2}g_{\alpha \beta }\left(r\right)dr \end{array}$$ (6)

where *CN* is the average number of a particular type of atom type *β* around *α* atomic site of another molecule found in a shell extending from *r*_1_ to *r*_2_ and *ρ*_*β*_ is the number density of atom type *β*. For different systems first solvation shell CNs are calculated by considering *r*_1_ = 0 and *r*_2_ = the position of first minimum of the corresponding pair-correlation function and the same are shown in Table 3.

**Table 3:** Number of first shell SCS molecules (*N*_*SCS*_), water molecules (*N*_*Water*_) and *γ*-tubulin protein residues (*N*_*Protein*−*Res*_) for different systems.

System	*N*_*SCS*_	*N*_*Water*_	*N*_*Protein*−*Res*_
P0			
P1		1.13	20.69
P2		0.86	21.22
P3		0.74	22.02
P4	3.14	6.70	10.38
P5	2.46	6.47	11.27
P6	2.32	6.06	12.62

As stated above, in systems P4-P6, we begin the simulation with a cluster of hydrotrope comprised of 24 SCS molecules and one griseofulvin drug molecule that reside inside the cage of the cluster. At the beginning of the simulation the number of first shell SCS molecules around drug molecule is 22 for all the systems P4-P6. From Table 3 it can be seen that for these systems the average number of hydrotrope molecules that are present in the first coordination shell of the drug griseofulvin are 3.14, 2.46 and 2.32 respectively. This implies that the drug molecule is being released from the cage of SCS clusters for systems P4-P6. Interestingly, though we have started with different initial configurations for systems P4-P6 but they exhibit almost similar results.

Now the question is after moving out from the cage of hydrotrope cluster whether griseofulvin molecule prefers to stay in the hydrophilic aqueous environment or in the binding pocket of *γ*-tubulin protein. To find out the answer of this question, we further determine the average number of first shell water molecules and average number of *γ*-tubulin residues around the *COM* of griseofulvin molecule. With the help of Eq 6, we have estimated the number of first shell water molecules as well as the average number of *γ*-tubulin residues that are present within 4 Å of drug molecule (see Table 3). We find that the numbers of first shell water molecules around the *COM* of griseofulvin are very low for the systems P1-P3 and in presence of hydrotrope molecules (systems P4-P6) these numbers increase moderately. Table 3 also depicts that in absence of SCS molecules the average number of protein residues around the drug molecule are 20.69, 21.22 and 22.02 for systems P1, P2 and P3 respectively, on the other hand for systems P4, P5 and P6 the same are 10.38, 11.27 and 12.68 respectively. These results clearly suggest that large number of protein residues are surrounding the drug molecule in absence of SCS molecules. A detailed comparison of the results indicate that the average number of protein residues around the griseofulvin is higher for the systems (P1-P3) that are devoid of hydrotrope molecules than the systems consist of hydrotrope SCS molecules (systems P4-P6). As in the initial configurations of the systems P4-P6, the drug containing hydrotrope cluster and *γ*-tubulin protein are in close contact, so the complete release of the drug molecule from the hydrotrope cluster is inhibited by the protein molecule. Moreover, since the distance between the drug and the protein in systems P4-P6 is relatively higher (due to encapsulation) than that for the systems P1-P3, the interactions of griseofulvin with possible binding residues of *γ*-tubulin protein is getting reduced for the former systems as a result of which number of protein residues around the drug griseofulvin molecule decreases. Nevertheless, we find that there are considerable number of protein residues in the first solvation shell of the drug molecules for the systems P4-P6. This clearly suggests that the drug griseofulvin once released from the hydrotrope encapsulation can interact with the protein residues.

In order to gather information about the possible binding pockets of *γ*-tubulin protein, we have considered the residues of *γ*-tubulin protein that are within 4 Å distance of griseofulvin. In an attempt to do so, we have implemented certain criteria. The cut-off distance between the *COM* of the griseofulvin molecule and any heavy atom of a residue of the protein should be within 4 Å and simultaneously, the residence time of a residue of *γ*-tubulin protein (which is lying within 4 Å distance of the griseofulvin molecule) should be more than 2.5% of the production phase trajectory. Table 4 shows the residues that fulfill these two criteria simultaneously for systems P1-P6. Residence times of different residues allow us to compare the most probable binding sites of *γ*-tubulin protein with griseofulvin. Note that, Efferth et al. [7] did the molecular docking study of *γ*-tubulin protein and griseofulvin drug molecules. They reported that the griseofulvin drug molecule binds with ASN-250 and LEU-360 residues of *γ*-tubulin protein. Other than this study, since there is no conclusive evidence of the binding sites, therefore, the simulations presented here constitute an extensive search for griseofulvin binding sites of *γ*-tubulin protein. We have found multiple binding sites of *γ*-tubulin protein for griseofulvin drug in different systems. In system P1, griseofulvin binds to H8, H9 helices and S7, S8, S9, S11, S13, and S14 strands. Griseofulvin is also observed to bind with loops joining the H6 helix and S8 strand, H8 and H9 helices and H9 helix and S9 strand in system P1. Binding to H1, H8, H9 helices and S1, S6, S7, S8, S14 strands is also observed in system P2. As, shown in Table 4, griseofulvin can also bind with loops joining the H1 helix and S2 strand, H8 and H9 helices. In system P3, we notice that *γ*-tubulin protein contains a binding pocket surrounded by *β* sheets S1, S6, S7, S8, S9, S12, and S14, alpha helices H8 and H9 and loops joining the helices H8 and H9. Although systems P1, P2 and P3 possess different initial starting configurations but they show similar binding patterns. In system P4, binding sites of griseofulvin are three beta sheets S6, S7 and S8, one helix H9, and two loops joining the H8 and H9 helices and H9 helix and S9 strand. S11, S14 strands and H13 helix, and loops joining the S10 and S11 strands and H11 helix and S13 strand come in close contact with griseofulvin drug molecule in system P5. In system P6, the griseofulvin drug molecule makes a contact with S11, S14 strands and H13 helix, and loops joining the H9 helix and S9 strand, S10 and S11 strands and H11 helix and S13 strand. Here also we have found an agreement in the griseofulvin binding sites of *γ*-tubulin for systems P4, P5 and P6. It is interesting to note that though all the binding sites of griseofulvin on *γ*-tubulin protein are not the same in all the systems (P1-P6) but they possess major similarity. Moreover, here it is worth to mention that our simulated results are in accordance with molecular docking results published elsewhere [7]. We also find that griseofulvin binding sites are remarkably consistent with that for colchicine binding on *γ*-tubulin protein [55].

**Table 4:** Residues of *γ*-tubulin that are present within the 4 Å distances of griseofulvin molecule.

System	
P1	LEU-164 (56.83), GLN-166 (94.27), TYR-168 (22.99), ASP-199 (17.42), CYS-200 (99.98), SER-238 (90.27), THR-241 (86.48), LEU-242 (29.85), TYR-247 (26.13), MET-248 (2.63), ASN-250 (45.37), ASP-251 (60.43), LEU-252 (99.99), ILE-253 (24.60), LEU-255 (99.63), ILE-256 (99.99), LEU-259 (80.83), ILE-260 (80.22), HIE-266 (73.34), LEU-268 (99.99), ALA-318 (46.46), ILE-319 (41.64), LEU-320 (99.99), ILE-322 (93.86), ALA-358 (75.00), MET-377 (99.72), ASN-379 (42.32)
P2	ARG-2 (77.37), GLU-3 (98.44), ILE-4 (99.77), THR-6 (9.59), PHE-20 (93.29), VAL-49 (42.83), PHE-50 (98.78), GLU-132 (99.93), GLY-133 (92.89), PHE-134 (64.16), VAL-135 (99.91), LEU-164 (33.82), GLN-166 (99.95), THR-167 (61.85), TYR-168 (99.96), CYS-200 (28.25), LEU-201 (28.38), VAL-202 (99.96), MET-235 (99.31), SER-238 (99.93), THR-239 (99.96), THR-240 (21.73), THR-241 (97.61), LEU-242 (99.95), LEU-252 (99.97), LEU-255 (61.01), ILE-256 (83.44), LEU-259 (4.53), MET-377 (78.65)
P3	ILE-4 (41.06), THR-6 (91.92), PHE-20 (71.16), PHE-50 (99.95), VAL-135 (99.93), LEU-136 (4.36), CYS-137 (11.21), GLN-166 (97.86), TYR-168 (99.83), THR-167 (4.85), CYS-200 (53.85), VAL-202 (99.95), ILE-234 (99.98), MET-235 (99.85), SER-238 (99.98), THR-239 (99.97), THR-241 (48.08), LEU-242 (99.96), ASN-250 (5.01), LEU-252 (99.52), LEU-255 (99.76), ILE-256 (99.88), LEU-259 (74.47), LEU-268 (99.99), MET-269 (4.94), THR-270 (99.91), ILE-322 (4.23), LEU-375 (87.77), MET-376 (3.70), MET-377 (99.98), ALA-378 (3.40)
P4	ARG-2 (45.55), GLU-132 (42.73), LYS-162 (3.18), LYS-163 (3.09), LYS-164 (99.99), VAL-165 (4.28), GLN-166 (89.52), TYR-168 (52.96), ASP-199 (60.42), CYS-200 (83.06), LEU-252 (99.93), ILE-253 (97.97), GLY-254 (15.02), ILE-256 (76.07), ALA-257 (94.52), PRO-263 (64.81)
P5	ARG-310 (19.22), ASN-313 (6.09), HIE-314 (6.30), CYS-315 (98.40), ILE-317 (8.29), ALA-343 (4.20), ASN-346 (21.03), PHE-347 (25.49), ILE-348 (96.58), PRO-349 (99.70), TRP-350 (99.99), THR-381 (76.44), SER-382 (19.70), ASN-434 (64.15), HIE-435 (14.42), ALA-437 (97.68), THR-438 (99.15), ARG-439 (94.51), PRO-440 (97.88)
P6	PRO-261 (6.45), THR-262 (6.69), LEU-265 (72.37), ARG-310 (5.93), HIE-314 (20.94), CYS-315 (70.98), TYR-316 (7.13), ILE-317 (99.96), ALA-343 (5.02), ALA-345 (88.08), ASN-346 (95.07), PHE-347 (91.09), ILE-348 (99.90), PRO-349 (83.56), TRP-350 (81.19), ASN-379 (9.88), THR-381 (92.66), SER-382 (6.08), ALA-437 (86.33), THR-438 (97.14), ARG-439 (80.64), PRO-440 (88.80)

Values in the parentheses represent the percentage of residence time of the corresponding residues of *γ*-tubulin protein around the drug.

**Fig 6:**
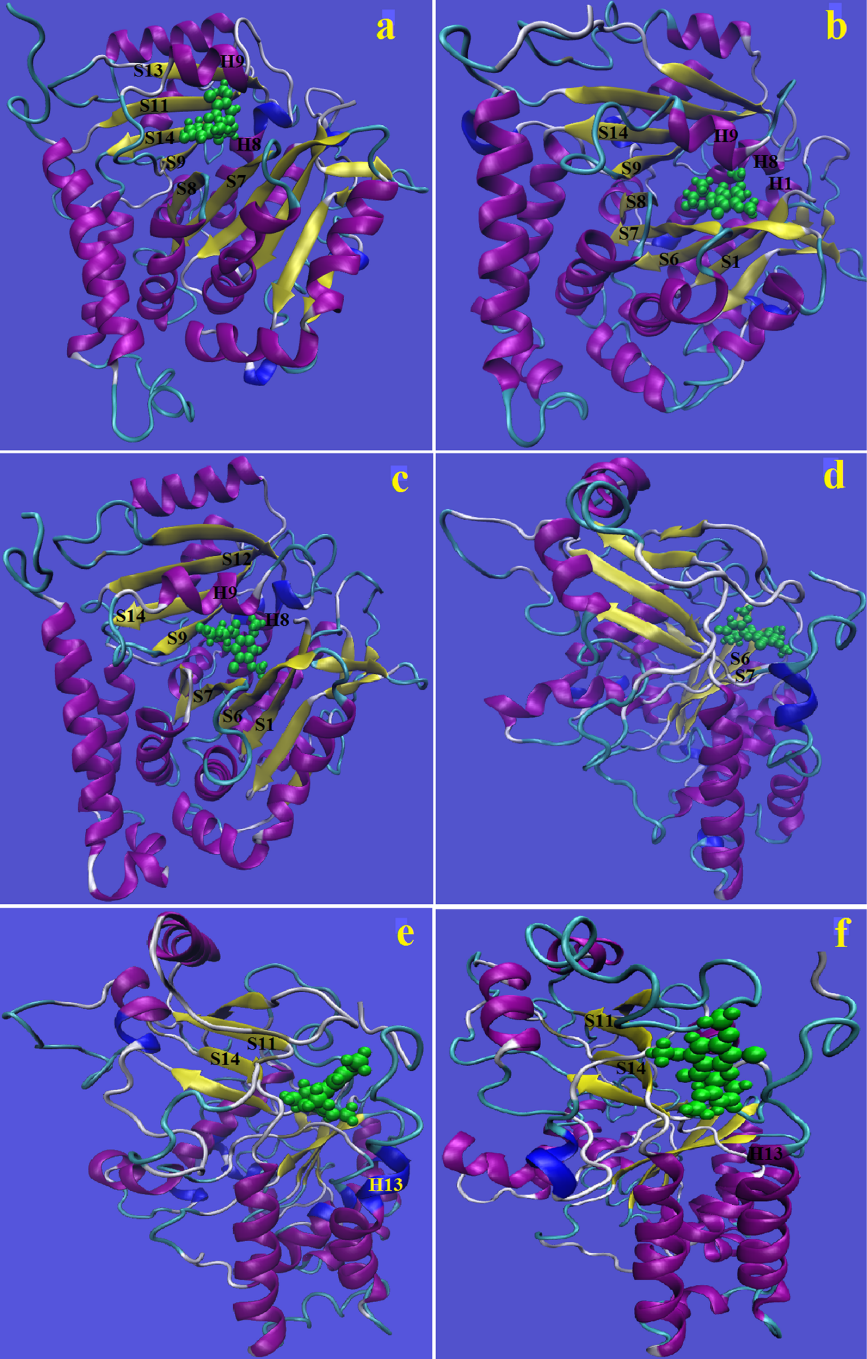
Binding motif of griseofulvin in *γ*-tubulin. The drug-protein complex is created by averaging the last 12 ns of simulated trajectory. (a) System P1, (b) system P2, (c) system P3, (d) system P4, (e) system P5 and (f) system P6.

Binding sites are also identified by constructing visualizing snapshots, averaged over frames from the last 12 ns of the simulation (see Fig 6). In system P1 (see Fig 6 (a)), the most probable contact residues lies in H8, H9 helices, S7, S8, S9, S11, S13, S14 strands and loops joining the H6 helix and S8 strand, H8 and H9 helices and H9 helix and S9 strand. From Fig 6 (b) it can be seen that griseofulvin’s most preferable location is in a core that is surrounded by H1, H8, H9 helices and S1, S6, S7, S8, S14 strands. Moreover, the loops joining the H1 helix and S2 strand, H8 and H9 helices of the *γ*-tubulin are in close contact with griseofulvin drug molecule. In system P3, the griseofulvin is found to occupy almost the similar position in *γ*-tubulin protein cage that we have found in systems P1 and P2. Fig 6 (d) shows that S6, S7 and S8 strands H9 helix and loops joining the H8 and H9 helices and H9 helix and S9 strand are in close contact with griseofulvin drug molecule. In system P5, contact residues located in S11, S14 strands and H13 helix, and loops joining the S10 and S11 strands and H11 helix and S13 strand. Fig 6 (f) displays that the binding sites for griseofulvin in system P6 are consistent with that for system P5.

### Hydrogen bond properties

To obtain more direct information about the interactions between the drug and protein binding sites, we have carried out hydrogen bond analysis between these two moieties. Our analysis reveals the key amino acids at the active binding centers of *γ*-tubulin protein with which griseofulvin forms hydrogen bonds. Only those hydrogen bonds are included in the Table 5 whose occupancy is more than 1%. The analysis shows that in absence of hydrotrope molecules (i.e., systems P1-P3) griseofulvin bounds with GLN-166 and SER-238 residues of *γ*-tubulin protein through hydrogen bonding interactions. In system P1, griseofulvin forms three hydrogen bonds with GLN-166 and one hydrogen bond with SER-238 residue. Of the four hydrogen bonds, the carbonyl group that is attached to five membered heterocyclic ring of griseofulvin drug molecule forms the most stable hydrogen bonds (occupancy 34.68%). In system P2 and P3, griseofulvin also form four hydrogen bonds with GLN-166 and SER-238 residues but they exhibit relatively low occupancy. A close examinations of these results reveal that it is mainly the GLN-166 residue of *γ*-tubulin protein which shows maximum hydrogen bonding interactions ability with griseofulvin drug molecules in absence of hydrotrope SCS molecules. In contrast griseofulvin forms hydrogen bonds with multiple residues of *γ*-tubulin protein in presence of hydrotrope SCS molecules (systems P4-P6). Moreover, these hydrogen bonds display very low occupancy. Only in system P6, griseofulvin forms moderately stable hydrogen bonds with THR-381 residue of *γ*-tubulin. These results further infer that hydrotrope molecules hinder the hydrogen bonding interactions and weaken the polar interactions between the *γ*-tubulin protein and griseofulvin drug molecule.

**Table 5:** Hydrogen bonding interactions between griseofulvin (GSV) and different residues of *γ*-tubulin protein.

System	Hydrogen Bonding Interactions			
	Atoms Involved 1-2-3	Bond Distance	Bond Angle	Occupancy
P1	GSV-O2-HE22-NE2-GLN-166	2.916	31.82	34.68
	GSV-O4-HE21-NE2-GLN-166	3.164	47.94	9.79
	GSV-O2-HE21-NE2-GLN-166	2.961	35.16	9.14
	GSV-O4-HG-OG-SER-238	3.224	46.78	1.14
P2	GSV-Cl-HE22-NE2-GLN-166	3.274	43.20	18.24
	GSV-Cl-HE21-NE2-GLN-166	3.233	41.58	11.95
	GSV-O1-HE21-NE2-GLN-166	3.249	46.71	5.46
	GSV-O2-HG-OG-SER-238	3.038	32.89	2.23
P3	GSV-O6-HG-OG-SER-238	3.177	36.73	4.32
	GSV-O2-HG-OG-SER-238	2.909	30.83	3.86
	GSV-O4-HG-OG-SER-238	3.159	34.03	1.72
	GSV-O1-HE21-NE2-GLN-166	3.142	38.13	1.16
P4	GSV-O5-HE22-NE2-GLN-166	2.962	37.98	4.32
	GSV-O5-HE21-NE2-GLN-166	2.995	36.95	1.64
	GSV-O2-HE21-NE2-GLN-166	3.195	45.79	1.09
P5	GSV-O3-HD21-ND2-ASN-346	3.113	22.75	5.54
	GSV-O5-HE2-NE2-HIE-314	3.052	41.04	3.94
	GSV-Cl-HE1-NE1-TRP-350	3.293	53.32	2.46
	GSV-O5-HG1-OG1-THR-381	2.969	21.94	1.06
P6	GSV-O3-HG1-OG1-THR-381	3.115	36.29	10.35
	GSV-Cl-HG1-OG1-THR-381	3.304	36.03	8.99
	GSV-O5-HD21-ND2-ASN-346	3.029	31.27	4.54
	GSV-O5-HG-OG-SER-382	3.358	59.27	1.32
	GSV-O4-HE1-NE1-TRP-350	3.258	37.54	1.06

Bond distance (in Å) refers to donor-acceptor cut-off distance and the bond angle (in Degree) is donor—H—acceptor cut-off angle. Occupancy (in %) represents percentage of simulation time occupied by a particular hydrogen bond.

### Binding free energy

The binding free energy (Δ*G*_*bind*_) between *γ*-tubulin protein and griseofulvin for different systems are tabulated in Table 6. The binding energy between the drug and protein is more favorable for systems without hydrotrope than that containing hydrotropes. This supports our coordination number analysis discussed above. As the number of protein residues around the drug griseofulvin increases binding ability between protein and drug also increases. Moreover, the drug griseofulvin alters its binding motif to *γ*-tubulin in presence of SCS clusters. In systems P4-P6 as the protein and SCS cluster are in close contact, the complete release of drug from hydrotrope clusters is difficult. Therefore, the binding ability of griseofulvin to *γ*-tubulin protein is getting decreased for systems P4-P6. Remarkably, pure drug containing systems i.e systems P1-P3 show very similar binding energies and in presence of hydrotrope molecule i.e for systems P4-P6 the binding energies between the *γ*-tubulin protein and griseofulvin are also close to each other.

**Table 6:** Binding free energies (Δ*G*_*bind*_) of *γ*-tubulin protein with griseofulvin for different systems.

System	Δ*E*_*vdw*_	Δ*E*_*ele*_	Δ*G*_*GB*_	Δ*G*_*NP*_	Δ*G*_*bind*_
P1	-45.09	-9.36	16.36	-4.95	-43.04
P2	-47.33	-11.32	20.08	-5.75	-44.32
P3	-51.39	-11.14	20.80	-5.65	-47.38
P4	-31.22	-3.10	11.92	-2.89	-25.29
P5	-32.28	-2.12	11.00	-3.19	-26.59
P6	-32.97	-2.93	11.66	-3.63	-27.87

Δ*E*_*vdw*_ and Δ*E*_*ele*_ are the van der Waals and electrostatic energies respectively, whereas Δ*G*_*GB*_ and Δ*G*_*NP*_ represent the polar and non-polar solvation free energies respectively. All values are in kcal mol^−1^ unit.

Table 6 also lists the values of the components of the binding free energy (Δ*G*_*bind*_). The van der Waals energy component (Δ*E*_*vdw*_) of drug-protein interactions makes the maximum contribution to the binding free energy. The electrostatic energy (Δ*E*_*ele*_) and the nonpolar solvation free energy (Δ*G*_*NP*_) are also favorable for binding. On the contrary the polar component of the solvation free energy (Δ*G*_*GB*_) is unfavorable for binding. Although, both van der Waals and electrostatic energy components play significant roles in binding free energy but it is mainly the hydrophobic contacts between the protein residues and the drug that make the binding favorable. As the number of first shell hydrotrope molecules around griseofulvin increases, the hydrophobic contacts between protein and drug also decreases. It explains the reason behind the lower (less favorable) van der Waals interaction energy for systems P4-P6 compared to that for systems P1-P3. Interestingly, the contribution Δ*E*_*ele*_ to Δ*G*_*bind*_ for hydrotrope containing systems P4-P6 is negligible. This finding acts as a corroborative evidence of what we have observed in the calculations of drug-protein hydrogen bonds discussed above.

## Summary and conclusions

In this study, we have investigated the drug binding ability and drug binding mechanism of *γ*-tubulin protein in presence and in absence of hydrotrope molecules using all atom MD simulations. Results give the details of structural changes of *γ*-tubulin protein due to interactions with hydrotrope or binding with drug molecule. Details analysis of the results suggests that the *γ*-tubulin proteins are more flexible in presence of hydrotrope molecules than that devoid of hydrotropes. Remarkably, in absence of hydrotrope molecules the conformational stability of protein is almost same like that in pure aqueous solution. Radius of gyration and SASA analysis also showed that *γ*-tubulin protein achieved more stable conformation in absence of hydrotrope molecules. The most noticeable changes in *γ*-tubulin protein, caused due to binding of drug griseofulvin, is observed in the functionally relevant loops of the protein. RMSF and average structure analysis showed that loops joining the S7 strand and H6 helix, H8 and H9 helices, S9 strand and H10 helix, S10 and S11 strands, H11 helix and S13 strand, and S13 and S14 strands undergo the largest conformational changes. These loops are crucial for binding of griseofulvin drug molecules. From the analysis of coordination number it can be inferred that the hydrotrope SCS cage does not prevent the drug from interacting with the protein. Further, these analyses also implies that the number of protein residues surrounding the griseofulvin drug molecule is higher in absence of hydrotrope molecules, indicating the stronger drug receptor interactions. The residues around the griseofulvin in different systems also predict the possible binding pockets of drug molecule. We further find more information about the binding pockets and binding motifs of griseofulvin with *γ*-tubulin protein from hydrogen bond analysis. The calculations of MM-GBSA suggest that the van der Waals, electrostatic and non polar solvation free interactions play important roles in the binding of griseofulvin with *γ*-tubulin protein for all the systems considered in this study. But, it is the drug-protein van der Waals interaction, which plays a dominant role. We find that the initial distance between the protein and drug could affect their binding interactions and the hydrotrope cluster also causes weakening of the drug-protein interactions. Thus, our results clearly demonstrate that the drug-protein interaction is not affected much in presence of SCS molecules. The only effect that one could see due to the encapsulation is that, the presence of SCS cluster in the starting configurations for systems P4-P6 causes the binding of the drug to different binding pockets of the protein when compared to systems without SCS clusters (systems P1-P3). Bearing in mind that there is no conclusive definitive answer for the possible binding sites of the protein *γ*-tubulin for the drug griseofulvin. In our opinion, proper systematic experimental works must be carried out in order to identify the actual binding sites of the protein for the drug griseofulvin. Remember that the drug griseofulvin used in this study is sparingly soluble in water and in our previous study we showed the enhancement in its solubility in water in presence of hydrotrope SCS [39]. Thus, the results presented in this article can definitely shed some lights on how does the encapsulation alter the drug-protein interactions. Nevertheless, our study not only provides molecular level mechanism and binding affinities of griseofulvin drug molecule in different systems but also delivers a detailed insight about the drug releasing ability of hydrotropic clusters. The results presented here could help in the drug designing and drug delivery approaches in future.

## Supporting information

S1 Fig Snapshots for 24:1 SCS-griseofulvin mixture in vacuum (a and b) and in presence of water (c and d). a and c are taken at the beginning of the simulation whereas b and d are captured at the end of the simulation. Pink balls represent griseofulvin molecules. Water molecules are left off for better visual clarity.
(ZIP)
