# Supplementary material for: Exploring the binding sites and binding mechanism for hydrotrope encapsulated griseofulvin drug on γ-tubulin protein
Source: PLoS One. 2018 Jan 11;13(1):e0190209. doi: 10.1371/journal.pone.0190209 (PMC5764265; doi:10.1371/journal.pone.0190209)
Supplement: S1 Fig — a and c are taken at the beginning of the simulation whereas b and d are captured at the end of the simulation. Pink balls represent griseofulvin molecules. Water molecules are left off for better visual clarity. (ZIP) [file pone.0190209.s001.zip › supportin_new.pdf]

Supporting Information

Exploring the binding sites and binding mechanism for hydrotrope encapsulated griseofulvin drug on γ-tubulin protein

Shubhadip Das and Sandip Paul*

Department of Chemistry, Indian Institute of Technology, Guwahati Assam, India-781039

(Dated: December 15, 2017)

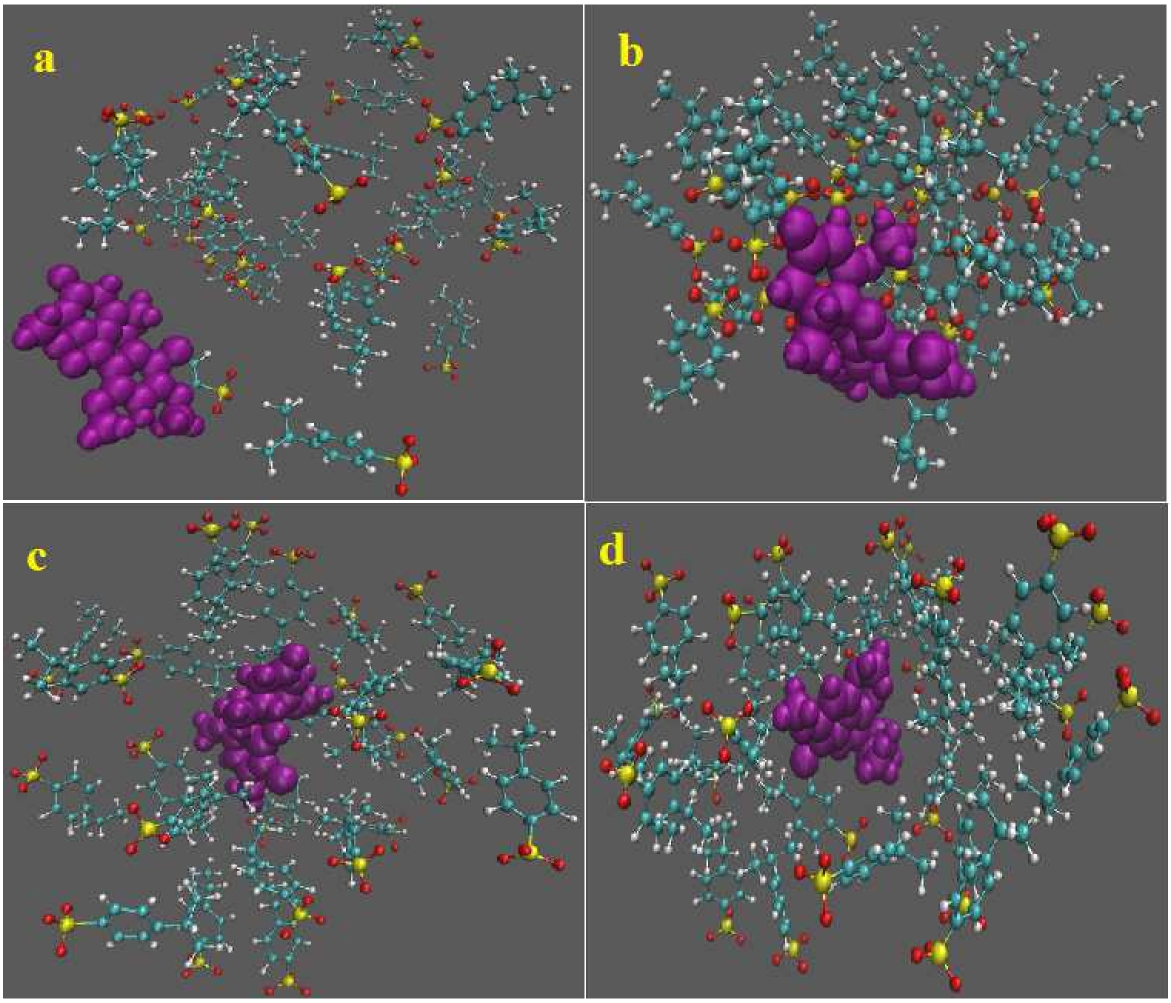

FIG. S1: Snapshots for 24:1 SCS-griseofulvin mixture in vacuum (a and b) and in presence of water (c and d). a and c are taken at the beginning of the simulation whereas b and d are captured at the end of the simulation. Pink balls represent griseofulvin molecules. Water molecules are left off for better visual clarity.

---

* Electronic address: sandipp@iitg.ernet.in
